# Trends in the Exposure of Nonsmokers in the U.S. Population to Secondhand Smoke: 1988–2002

**DOI:** 10.1289/ehp.8850

**Published:** 2006-02-02

**Authors:** James L. Pirkle, John T. Bernert, Samuel P. Caudill, Connie S. Sosnoff, Terry F. Pechacek

**Affiliations:** 1 Division of Laboratory Science, National Center for Environmental Health and; 2 Office of Smoking and Health, National Center for Chronic Disease Prevention and Health Promotion, Centers for Disease Control and Prevention, Atlanta, Georgia, USA

**Keywords:** biomarker, cotinine, environmental tobacco smoke, ETS, health and nutrition examination survey, NHANES, secondhand smoke, SHS, tandem mass spectrometry

## Abstract

The objective of this study was to describe the exposure of nonsmokers in the U.S. population to secondhand smoke (SHS) using serum cotinine concentrations measured over a period of 14 years, from October 1988 through December 2002. This study consists of a series of National Health and Nutrition Examination Surveys (NHANES) measuring serum cotinine as an index of SHS exposure of participants. Study participants were individuals representative of the U.S. civilian, noninstitutionalized population, ≥ 4 years of age. We analyzed serum cotinine and interview data from NHANES obtained during surveys conducted during four distinct time periods. Our results document a substantial decline of approximately 70% in serum cotinine concentrations in non-smokers during this period. This decrease was reflected in all groups within the population regardless of age, sex, or race/ethnicity. The large decrease that we observed in serum cotinine concentrations suggests a substantial reduction in the exposure of the U.S. population to SHS during the 1990s. The exposure of nonsmokers to SHS represents an important public health concern. Our findings suggest that recent public health efforts to reduce such exposures have had an important effect, although children and non-Hispanic black nonsmokers show relatively higher levels of serum cotinine.

Health risks associated with the active use of tobacco have been documented extensively over many years. After the first Surgeon General’s report on smoking and health in 1964, the prevalence of cigarette smoking in the United States began a gradual decline [Department of Health and Human Services (DHHS) 2000a], although the use of tobacco continues to be an important problem and remains the leading preventable cause of death and disability in the United States (DHHS 2004). In addition to mainstream smoke that is inhaled by the smoker, burning cigarettes also generate secondhand smoke [SHS; also sometimes referred to as environmental tobacco smoke (ETS)] that is formed from smoke emitted into the environment from the smoldering tip of the cigarette, mixed with smoke exhaled by the smoker [National Research Council (NRC) 1986]. Involuntary smoking results when nonsmokers are exposed to SHS, and health risks for nonsmokers posed by involuntary smoking were gradually realized. As early as 1972, the topic of SHS and the potential risk faced by nonsmokers exposed to SHS were noted in a Surgeon General’s report addressing the use of tobacco (DHHS 1972). An important further impetus for investigations regarding adverse health effects from SHS exposure resulted from the 1986 Surgeon General’s report (DHHS 1986), which for the first time focused on the health risks of SHS, and also from two influential reports by the National Research Council (NRC 1986) and the U.S. Environmental Protection Agency (U.S. EPA 1992). Both reports concluded that exposure to SHS causes lung cancer in nonsmokers and has other adverse effects in both adults and children. Several subsequent reports have confirmed and extended this link between SHS and adverse health effects (Jaakola and Samet 1999; National Cancer Institute 1999), which may include cancer, asthma, respiratory infections, decreased pulmonary function, and cardiovascular disease.

Despite the increasing awareness that SHS represents an important public health concern, the extent of the problem was initially difficult to measure because data on the exposure of nonsmokers were limited and often depended solely on self-reported exposure or on inferences, such as living with a smoker, rather than on direct measurements. However, objective biomarkers of exposure to tobacco have been identified and validated (Benowitz 1983, 1996; Jarvis et al. 1988), and an expert panel convened to review the prospects for biomarker measurements as an index of SHS exposure concluded that plasma cotinine was the marker of choice (Watts et al. 1990). Cotinine, the primary proximate metabolite of nicotine, is specific for exposure to tobacco, and it is preferred as a marker over nicotine itself partly because the half-life of cotinine in the body of about 18 hr (Benowitz 1983; Jarvis et al. 1988) is much longer than that of nicotine. Serum cotinine can mark the exposure of an individual to tobacco only over the previous few days, and it is subject to interindividual variations in the metabolism of nicotine. However, these limitations are not substantial drawbacks when comparing mean values from groups of people, and in a review, Benowitz (1996) concluded that the evidence supports cotinine measurements providing “a valid and quantitative measure of average human ETS exposure over time.”

The first national survey of SHS exposure of the entire U.S. population based on serum cotinine measurements was conducted as part of the Third National Health and Nutrition Examination Survey (NHANES III) that covered the time period of 1988–1994. NHANES III consisted of two phases, and we previously reported the results of cotinine measurements conducted with > 10,000 participants ≥ 4 years of age from phase 1, extending from 1988 through 1991 (Pirkle et al. 1996). Our results at that time indicated widespread exposure of the population to tobacco smoke. Overall, 88% of nonsmokers in the U.S. population in that study were found to have detectable levels (≥ 0.050 ng/mL) of cotinine in their blood, and certain groups of nonsmokers, including blacks, males, and children, were found to be at elevated risk of exposure based on their serum cotinine levels (Pirkle et al. 1996).

After phase 1, additional data were acquired during the continuation of NHANES III in phase 2, which extended from 1991 through 1994. No further studies were conducted during 1995–1998, but NHANES resumed in 1999 and has been continuous from that time onward, providing a new sampling of the U.S. population every 2 years. Serum cotinine was measured in NHANES III (1988–1994), in NHANES 1999–2000, and NHANES 2001–2002. Thus, we now have acquired data from NHANES for > 10 years that represent exposures after our initial report from the time period 1988–1991 (Pirkle et al. 1996). We report here the analysis of these data extending from 1988 through 2002, which indicates a decreasing trend in SHS exposure of nonsmokers in the United States, most likely reflecting extensive efforts made by the public health community during this time to reduce smoking in the home and the exposure of nonsmokers in public places. However, our results also indicate that two groups in the population, blacks and children, show relatively higher levels of SHS exposure during this time, suggesting that further work should provide special focus on these at-risk groups.

## Materials and Methods

NHANES is a survey conducted by the National Center for Health Statistics, Centers for Disease Control and Prevention (CDC), which is designed to examine a nationally representative sample of the U.S. civilian, noninstitutionalized population based on a complex, stratified, multistage probability cluster sampling design. The protocols included a home interview followed by a physical examination in a mobile examination center (MEC). These studies were approved by the National Center for Health Statistics Institutional Review Board, and all subjects (or their parent or guardian) provided informed consent before participation. During examination in the MEC, blood samples were drawn for serum cotinine analysis from all participants ≥ 4 years of age during NHANES III, and from participants ≥ 3 years of age in subsequent surveys. However, to maintain comparability among surveys in our present analyses, we have included only participants ≥ 4 years of age in each study interval.

NHANES III consisted of two phases, phase 1 extending from 1988 to 1991, and phase 2 from 1991 through 1994. In 1999, NHANES began operation on a continual basis, providing a new sampling of the U.S. population every 2 years. The data reported here were acquired during NHANES III, phases 1 and 2, and in NHANES 1999–2000 and NHANES 2001–2002. Thus, they cover four distinct intervals within an overall time period of 14 years, from 1988 through 2002.

### Participants

Nonsmokers were defined in this study as persons whose serum cotinine concentration was ≤ 10 ng/mL. Previous comparisons in NHANES III demonstrated little difference when cutoffs of either 10 or 15 ng/mL were used (Pirkle et al. 1996). Race/ethnicity of the participants based on self-report was categorized as non-Hispanic white, non-Hispanic black, Mexican American, or other. The race/ethnicity category of “other” was included in mean and percentile estimates for the total population, but not in the regression models when race was included as a covariate. Age was the age in years at the time of interview. A total of 29,849 participants were included in this study.

### Cotinine analysis

We measured serum cotinine by a high-performance liquid chromatography/atmospheric-pressure ionization tandem mass spectrometry (LC/MS/MS) method that has been described previously (Bernert et al. 1997, 2000). Briefly, serum samples were equilibrated with a trideuterated cotinine internal standard and then extracted using ChemElute columns (Varian, Harbor City, CA), concentrated, and analyzed by LC/MS/MS using atmospheric pressure chemical ionization. The limit of detection (LOD) for most of these analyses was 0.050 ng/mL in serum, and we periodically evaluated the results to assure that sensitivity at this level was maintained. However, as a result of continuing method improvements, including the introduction of a newer, more sensitive mass spectrometer, the LOD was lowered to 0.015 ng/mL during the analysis of samples from NHANES 2001–2002. Approximately 85% of the samples from 2001–2002 were analyzed using the newer, more sensitive LOD. For comparison, we also analyzed the 2001–2002 samples using an LOD of 0.050 ng/mL and found little difference in estimates for means and percentiles.

This LC/MS/MS method for serum cotinine has been continuously maintained in a single laboratory at the CDC and has analyzed NHANES samples collected since 1988. Both bench and blind serum pools are routinely included with each analytic run as part of the quality assurance program for this method, and additional pools spiked with known amounts of cotinine perchlorate are analyzed periodically to confirm both precision and accuracy of the assay. Because of ongoing improvements in the methodology and because of the potential for monitoring population trends in exposure, we have made a particular effort to assure continuity, stability, and uniformity in our analyses over an extended time period. As part of that effort, residual samples are retained from older serum quality control pools when new pools are prepared, and aliquots of the older pools are periodically reexamined to help confirm stability of the method. We have previously noted that serum cotinine is stable when stored at –60°C (Bernert et al. 2000). Three pools at levels of 0.268 ng/mL, 1.86 ng/mL, and 207 ng/mL have been measured periodically from 1990 through 2004 in this manner, and those data confirmed that no systematic drift has occurred in our measurement of serum cotinine during this time.

### Statistical methods

All regression models were fitted using SUDAAN PROC REGRESS (Research Triangle Institute, Research Triangle Park, NC). All analyses incorporated sampling weights that adjusted for unequal probabilities of selection. The log of serum cotinine was used as the dependent variable because of the log-normal distribution of serum cotinine that has been previously described (Pirkle et al. 1996). Separate models were fitted for each time period. For each of the models, the following independent variables were used: sex (males, females), race/ethnicity (non-Hispanic whites, non-Hispanic blacks, Mexican Americans), and age group (4–11, 12–19 and ≥ 20 years). Initial models also included all possible two-way interactions. Geometric means computed for each age group, race/ethnicity, and sex were adjusted for all other variables in each model. Changes over time were evaluated by using a two-tailed *t*-test to compare model adjusted means of the log-transformed results for the periods 1988–1991 and 1999–2002. The degrees of freedom and SEs for these tests correspond to those associated with the model-adjusted means. In all cases, a null hypothesis probability level of ≤ 5% was taken to indicate statistical significance.

## Results

**Figure 1:**
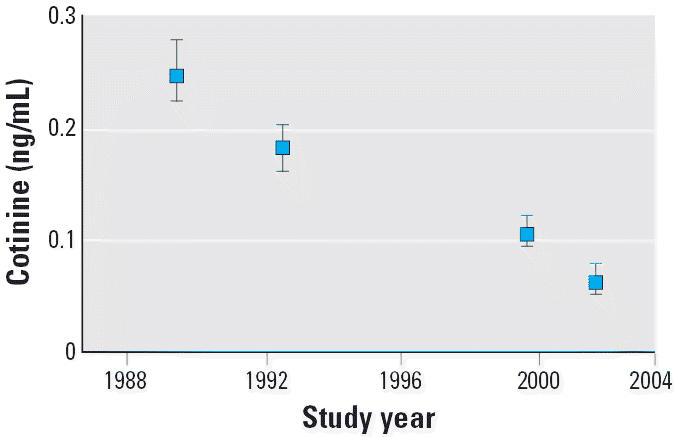
Serum cotinine geometric means (95% CI) for U.S. nonsmokers by study interval: exposure of nonsmokers in the U.S. population to SHS, 1988–2002. The data are plotted at the approximate midpoint for four separate time intervals: 1988–1991 (NHANES III, phase 1), 1991–1994 (NHANES III, phase 2), 1999–2000, and 2001–2002.

Figure 1 shows the geometric mean [and 95% confidence interval (CI)] for serum cotinine concentrations in the U.S. population of nonusers of tobacco over the time period 1988–2002. The four time periods included in this and subsequent figures are 1988–1991, 1991–1994, 1999–2000, and 2001–2002; the data in Figure 1 are plotted at the approximate midpoint of each interval. These data demonstrate a consistent decrease over time, with a decline of approximately 70% observed between the first and last survey periods. In NHANES III, phase 1, nearly all nonsmokers (88%) had concentrations of cotinine ≥ 0.050 ng/mL in their blood (Pirkle et al. 1996), a proportion that decreased to 80% in NHANES III, phase 2. The proportion of the population with cotinine concentrations ≥0.050 ng/mL further decreased to 51% in NHANES 1999–2000 and to 43% in NHANES 2001–2002.

**Figure 2:**
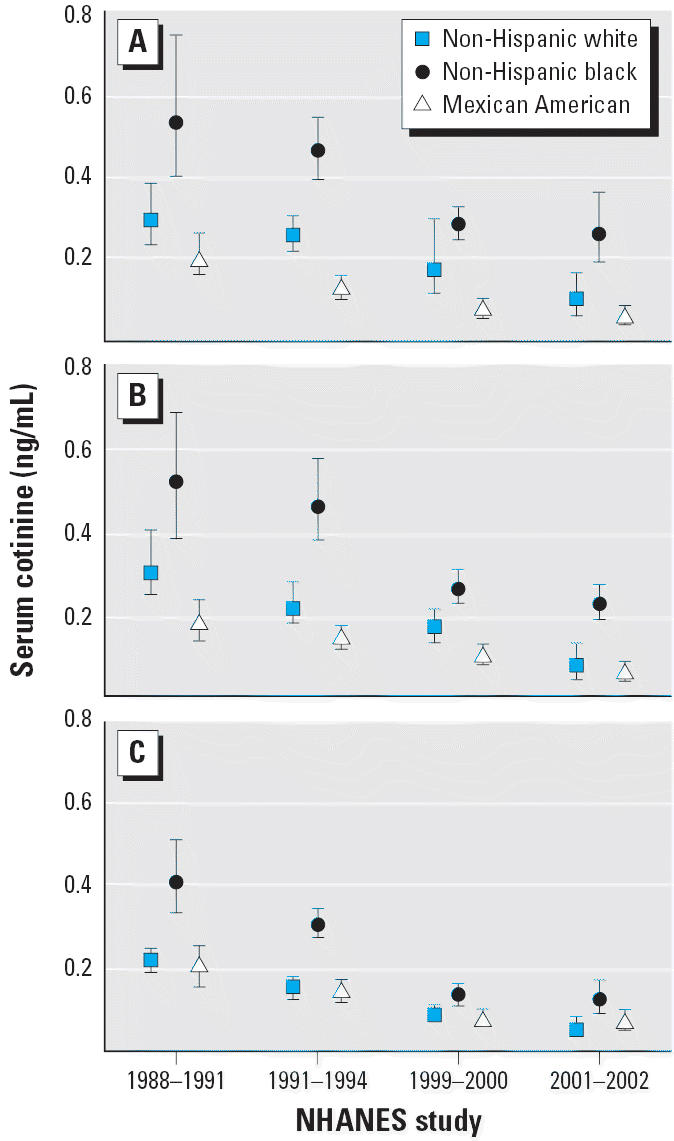
Serum cotinine adjusted geometric means (95% CI) by age and race/ethnicity—exposure of nonsmokers in the U.S. population to SHS, 1988–2002: (*A*) 4–11 years of age, (*B*) 12–19 years of age, and (*C*) ≥ 20 years of age. The data are plotted at the approximate midpoint for each of the four separate time intervals, with the individual race/ethnicity groups offset for clarity.

**Figure 3:**
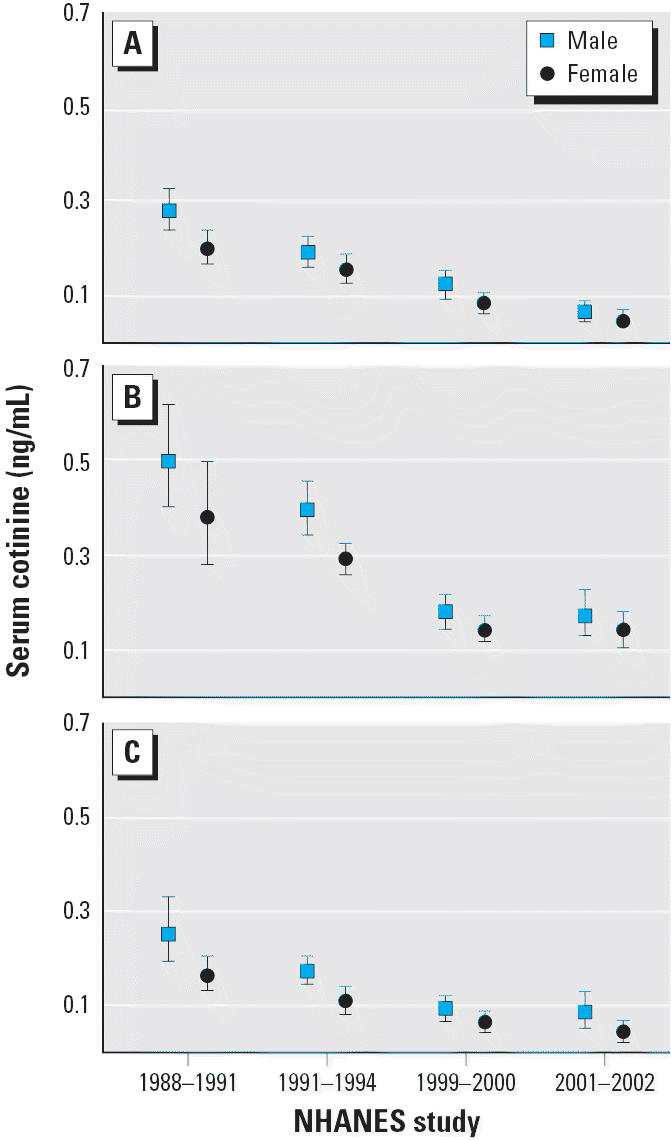
Serum cotinine adjusted geometric means (95% CI) by race/ethnicity and sex—exposure of nonsmokers in the U.S. population to SHS. 1988–2002: (*A*) non-Hispanic white, (*B*) non-Hispanic black, and (*C*) Mexican American. The data are plotted at the approximate midpoint for each of the four separate time intervals, with the two sex groups offset for clarity.

**Figure 4:**
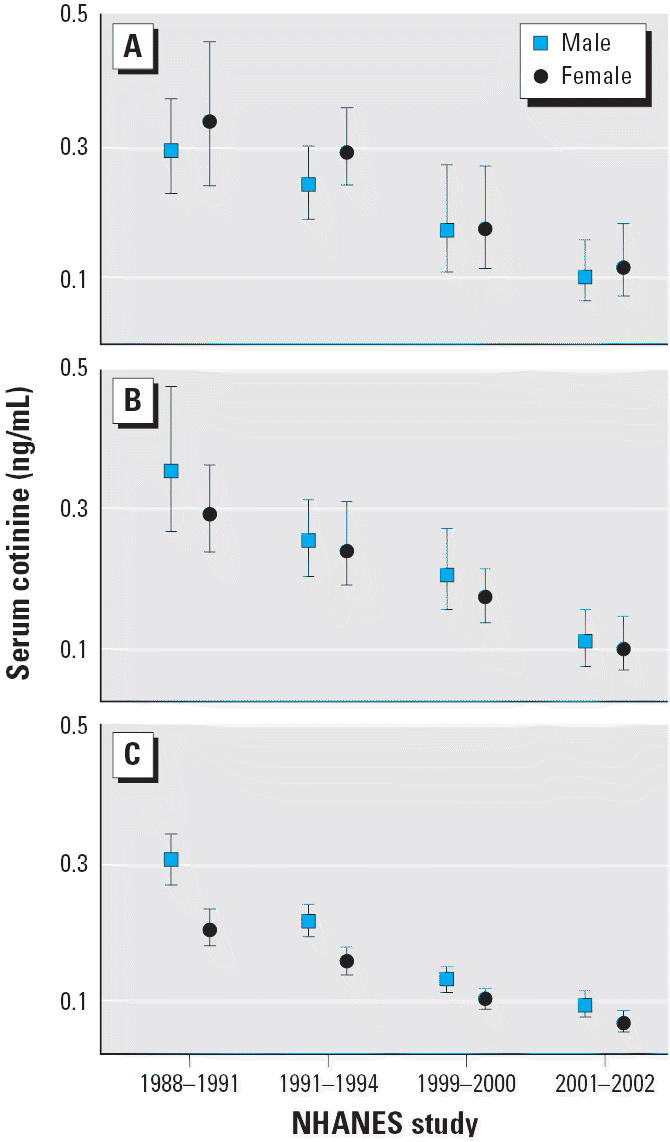
Serum cotinine adjusted geometric means (95% CI) by age and sex—exposure of non-smokers in the U.S. population to SHS, 1988–2002: (*A*) 4–11 years of age, (*B*) 12–19 years of age, and (*C*) ≥ 20 years of age. The data are plotted at the approximate midpoint for each of the four separate time intervals, with the two sex groups offset for clarity.

Trends in the adjusted geometric mean cotinine concentrations (adjusted for age, race, and sex) for the population subdivided by age and race/ethnicity are given in Figure 2, by race/ethnicity and sex in Figure 3, and by age and sex in Figure 4. We evaluated changes over time by using *t*-tests to compare model-adjusted geometric means for time periods 1988–1991 with those for 1999–2002 (results not shown). Because sex × age and race × age interactions were present in those models for one or both time periods, we examined contrasts between the two time periods separately for each sex/age category and for each race/age category. A significant decrease in serum cotinine was observed comparing 1988–1991 with 1999–2002 for each sex/age and race/age category except for non-Hispanic whites 4–11 years of age (*p* = 0.066).

In general, cotinine concentrations in each survey were significantly higher in children than in adults among both non-Hispanic whites and non-Hispanic blacks. Mexican Americans were an exception, however, for which children’s values were not significantly higher than those of adults in any of the time intervals (Figure 2). Serum cotinine levels in NHANES also clearly differed by race/ethnicity. During each time period, the order for adjusted mean cotinine concentrations remained Mexican American < non-Hispanic white < non-Hispanic black, although the mean levels for Mexican-American and non-Hispanic white nonsmokers were not significantly different in the most recent (2001–2002) time period. Non-Hispanic blacks had mean cotinine concentrations significantly higher than those of either Mexican Americans or non-Hispanic whites during each time interval. The only exception was for non-Hispanic whites 4–11 years of age, for whom the difference from non-Hispanic blacks approached but did not achieve statistical significance (*p* = 0.059).

Figure 4 summarizes results by age and sex. A modest difference by sex was noted among adults ≥ 20 years of age, with men having significantly higher mean serum cotinine concentrations than did women in each time interval. Cotinine levels were also slightly higher in male adolescents (12–19 years of age) in every case, but those differences were not statistically significant. This pattern was reversed among the younger children in the 4–11 age group, with girls having consistently higher mean serum cotinine levels than did boys, although again, the differences were small and were not significant.

Percentiles for serum cotinine in non-smokers in three main age groups are given in Table 1. Median concentrations tended to be higher among children and adolescents in each interval, with the greatest differences between adults ≥ 20 years of age versus younger participants. In addition, the decreases in concentrations from 1988 to 2002 were much less evident among individuals with the greatest exposure. Among adults, levels denoting the 95th percentile decreased only about 40% during this time, whereas the 95th percentile values among children and adolescents remained virtually unchanged throughout the entire period from 1988 through 2002. Similar results were seen for the most highly exposed individuals among non-Hispanic blacks, where again the 90th and 95th percentiles showed little decrease for the entire time period from 1988 to 2002 (Table 2).

## Discussion

Comparison of nonsmoker serum cotinine concentrations acquired from NHANES over a period of 14 years clearly demonstrates a substantial decline, averaging approximately 70% overall, during this time. This decrease in serum cotinine concentrations suggests a substantial reduction in the exposure of the U.S. population to SHS over this period. For example, during NHANES III, approximately 65% of nonsmokers had serum cotinine concentrations > 0.1 ng/mL (DHHS 2000b). On that basis, a Healthy People 2010 objective was established stating that, by the year 2010, no more than 45% of nonsmokers should have cotinine levels > 0.1 ng/mL (DHHS 2000c). Our present results suggest that this goal was met during 1999–2000. However, children and non-Hispanic blacks had consistently higher serum cotinine concentrations than did other segments of the population and thus remain at relatively elevated risk from exposure to SHS.

The most likely explanations for this decrease in serum cotinine concentrations are the increased restrictions on smoking that have been widely instituted at work and in other public places during this time period, and further efforts to reduce the exposure of nonsmokers in the home. Using data from the Current Population Survey, Shopland et al. (2001) found that between 1993 and 1999 the percentage of indoor workers reporting smoke-free policies in the workplace increased from less than 46% to nearly 70%. In 1993, only two states had at least 60% of indoor workers reporting that smoke-free policies were in place; by 1999, 47 states (and the District of Columbia) had at least that level of coverage. In general, women reported more workplace smoke-free policies than did men, although the sex difference narrowed somewhat by 1999. Smoke-free workplaces are also known to contribute to a reduction in smoking prevalence among workers (Farrelly et al. 1999; Fichtenberg and Glantz 2002). Increases in smoke-free policies and reductions in smoking prevalence may both have contributed to the decline in exposure of non-smokers to SHS. The role of restrictions, however, is presumably more important because the prevalence of adult smoking in the United States did not decrease substantially during the 1990s: between 1990 and 1999, the national median decreased only slightly from 25.5% to 22.7% (CDC 2000). In general, smoke-free environmental policies are regarded as the most effective means of reducing exposure to SHS (Task Force on Community Preventive Services 2001).

Certain subgroups, when characterized by age, sex, or race/ethnicity, had consistently higher cotinine concentrations during each time period. A major source of SHS exposure in young children is from parents or other adults smoking at home, and exposure of non-smokers to SHS in homes with children also declined during the 1990s. Comparing data from the 1992 and 2000 National Health Interview Surveys, Soliman et al. (2004) found that reported SHS exposure declined from 36% to approximately 25%, more than would be expected from declines in adult smoking prevalence alone. This decrease occurred across all groups, although home SHS exposures remained most prevalent among non-Hispanic whites in that study, and we found that cotinine concentrations were higher in non-Hispanic children than in adults during all NHANES survey periods.

In the present study, Mexican-American nonsmokers generally had lower serum cotinine levels than did the other two race/ethnicity groups, whereas mean serum cotinine concentrations in non-Hispanic blacks were consistently higher in each time period. The higher cotinine concentrations found in non-Hispanic blacks presumably reflect greater exposure to SHS within this population, although the interpretation of these results is complicated by possible metabolic influences. Among active smokers, blacks have consistently higher serum cotinine concentrations per cigarette smoked than do whites (Caraballo et al. 1998; Wagenknecht et al. 1990), and this has been attributed, at least in part, to differences between blacks and whites in the metabolism of nicotine, cotinine, and their glucuronides (Benowitz et al. 1999). Thus, metabolic factors might also account for at least part of the ethnic/racial differences seen in serum cotinine levels among nonsmokers. In a study of children with asthma, Wilson et al. (2005) reported finding significantly higher serum cotinine concentrations for African-American children even after adjusting for self-reported SHS exposure. However, Wagenknecht et al. (1993) found that SHS exposure assessed by either self-report or serum cotinine measurements among 3,300 nonsmokers in the CARDIA study was significantly higher for blacks than for whites, but that the difference in serum cotinine did not persist after adjustment for self-reported exposure to SHS. Sexton et al. (2004) also found that questionnaires, time–activity data, and cotinine measurements all indicated higher SHS exposure among African-American children. Thus, the consistently higher serum cotinine levels for black nonsmokers in NHANES appear to reflect higher SHS exposure, although the extent to which differences in metabolism may confound these estimates remains uncertain.

Exposure of nonsmokers to SHS also appears to have declined in other countries besides the United States during this time period, at least based on self-report. For example, Borland et al. (1999) described annual surveys of approximately 2,500 adults conducted in Victoria, Australia, from 1989 to 1997. The percentage of respondents reporting that they did not smoke in the presence of children and that visitors were discouraged from smoking in the home both approximately doubled during this time. In addition, the percentage of indoor workers in Victoria protected by restrictions on smoking in the workplace increased from 17% to 66% between 1988 and 1995. Within 1 year after Finland passed its Tobacco Control Act in 1995 prohibiting smoking in the workplace in all joint and public premises, workers reporting no ETS exposure in the workplace increased almost 3-fold, from 19.2% to 54.2% (Heloma et al. 2001).

Most studies finding decreased exposure to SHS over time have relied on self-reports. However, Jarvis et al. (2000) have reported a substantial decrease during the 1990s in the exposure of British school children to SHS as assessed by salivary cotinine measurements. They monitored secondary school children 11–15 years of age from 1988 through 1996 and found that their salivary cotinine levels decreased by almost 50% during that time. They attributed the decline to both the decrease in prevalence of smoking among young adults with children and the increased restrictions in Great Britain on smoking in public places. However, Jarvis et al. (2000) found only small declines among children whose parents smoked, suggesting that cessation rather than smokers simply avoiding exposure of children in the home was the primary factor driving the decline.

Our study has several strengths and some limitations. The data were taken from several large surveys conducted over a period of 14 years, evaluating national samples of individuals who were representative of the entire U.S. civilian, noninstitutionalized population, and included a total of nearly 30,000 nonsmokers. We used a sensitive and specific method for serum cotinine analysis, and all assays were conducted under uniform and rigorously controlled conditions. Repetitive analyses of common samples over time confirmed the absence of any unusual variations or drift in the analytic method. Thus, the substantial decreases in serum cotinine we observed over time most likely reflect corresponding decreases in exposure of nonsmokers to SHS. Nevertheless, serum cotinine has limitations as an exposure marker because it can monitor exposures only over the previous few days, and because nicotine metabolic differences among groups may influence the concentrations observed. However, despite the relatively short half-life of cotinine, measures of central tendency among groups based on large numbers of individuals should provide reasonable estimates of group steady-state levels. Although the differences we observed among ethnic/racial groups most likely reflect differences in exposure, metabolic influences cannot be excluded, and additional work is needed to evaluate the relative contributions of exposure and metabolism. Finally, a few occasional smokers could possibly have been included inadvertently among the more highly exposed participants in our study, because some infrequent smokers may have serum cotinine levels < 10 ng/mL. This factor is unlikely to have been significant among adults, and it is even less likely to have been influential among young children because few children in the 4–11 age group are active smokers.

## Conclusion

Serum cotinine concentrations among non-smokers in the U.S. population declined significantly during the 1990s. This decrease was found in all groups within the population and probably reflects the substantial progress made in reducing the exposure of nonsmokers to SHS during this time. Nevertheless, children and non-Hispanic blacks continue to show relatively higher serum cotinine concentrations, suggesting that these two groups in particular should be the focus of increased intervention efforts, and that additional work is needed to further encourage restrictions on smoking in the home, automobiles, and other locations when children are present.

## Figures and Tables

**Table 1:** Percentile points for serum cotinine in nonsmokers by age.

	Selected percentile (95% CI)			
Age group	1988–1991	1991–1994	1999–2000	2001–2002
Children (4–11 years of age)				
50th percentile	0.262 (0.194–0.401)	0.211 (0.167–0.254)	0.110 (0.062–0.173)	0.067 (0.038–0.118)
75th percentile	1.01 (0.791–1.29)	0.935 (0.769–1.10)	0.489 (0.264–1.06)	0.495 (0.275–0.934)
90th percentile	2.16 (1.72–2.75)	2.40 (2.04–2.85)	1.82 (0.91–3.47)	2.03 (1.42–2.53)
95th percentile	3.33 (2.62–3.82)	3.58 (2.95–4.49)	3.44 (1.34–4.79)	3.05 (2.44–3.37)
No.	1,839	2,090	1,065	1,278
Adolescents (12–19 years of age)				
50th percentile	0.247 (0.204–0.340)	0.208 (0.152–0.250)	0.107 (0.080–0.160)	0.051 (0.032–0.109)
75th percentile	0.848 (0.672–1.17)	0.658 (0.530–0.999)	0.540 (0.428–0.660)	0.352 (0.189–0.580)
90th percentile	2.14 (1.66–2.62)	1.92 (1.75–2.34)	1.65 (1.48–1.92)	1.53 (1.09–2.12)
95th percentile	3.18 (2.48–4.23)	3.06 (2.49–3.29)	2.56 (2.09–3.39)	3.12 (2.47–3.99)
No.	1,094	1,418	1,773	1,902
Adults (≥ 20 years of age)				
50th percentile	0.204 (0.178–0.233)	0.128 (0.111–0.151)	0.035 (0.035–0.060)	0.034 (0.024–0.038)
75th percentile	0.522 (0.463–0.613)	0.353 (0.308–0.417)	0.167 (0.140–0.193)	0.113 (0.090–0.150)
90th percentile	1.35 (1.11–1.61)	0.948 (0.822–1.16)	0.630 (0.530–0.810)	0.623 (0.465–0.770)
95th percentile	2.37 (1.93–2.80)	1.73 (1.45–2.18)	1.48 (1.28–1.66)	1.38 (1.11–1.84)
No.	5,157	5,684	3,052	3,497

**Table 2:** Percentile points for serum cotinine in nonsmokers by race/ethnicity.

	Selected percentile (95% CI)			
Race/ethnicity	1988–1991	1991–1994	1999–2000	2001–2002
Non-Hispanic white				
50th percentile	0.199 (0.172–0.234)	0.129 (0.109–0.158)	0.050 (0.035–0.070)	0.035 (0.022–0.042)
75th percentile	0.573 (0.457–0.698)	0.384 (0.309–0.493)	0.210 (0.150–0.308)	0.115 (0.084–0.174)
90th percentile	1.48 (1.17–1.88)	1.30 (0.97–1.61)	0.916 (0.621–1.28)	0.755 (0.533–1.06)
95th percentile	2.50 (2.00–2.97)	2.39 (1.92–2.86)	1.85 (1.35–2.74)	1.81 (1.41–2.23)
No.	3,150	3,023	1,926	2,798
Non-Hispanic black				
50th percentile	0.458 (0.326–0.608)	0.338 (0.286–0.394)	0.130 (0.110–0.144)	0.130 (0.105–0.159)
75th percentile	1.16 (0.943–1.44)	0.942 (0.825–1.06)	0.493 (0.390–0.599)	0.556 (0.436–0.749)
90th percentile	2.43 (2.05–2.84)	2.06 (1.87–2.28)	1.39 (1.14–1.66)	1.74 (1.54–1.98)
95th percentile	3.46 (3.14–4.04)	3.08 (2.78–3.46)	2.26 (1.78–3.19)	3.01 (2.42–3.78)
No.	1,850	2,871	1,303	1,557
Mexican American				
50th percentile	0.173 (0.136–0.221)	0.101 (0.080–0.125)	—	0.036 (0.025–0.060)
75th percentile	0.437 (0.327–0.559)	0.314 (0.246–0.380)	0.136 (0.110–0.170)	0.157 (0.080–0.308)
90th percentile	1.13 (0.874–1.52)	0.904 (0.890–1.14)	0.506 (0.372–0.738)	0.670 (0.417–1.19)
95th percentile	2.33 (1.49–3.56)	1.89 (1.42–2.47)	1.24 (0.900–1.71)	2.06 (1.14–2.96)
No.	2,807	2,794	2,196	1,843
